# Investigation of Friction Stir Welding of Additively Manufactured Biocompatible Thermoplastics Using Stationary Shoulder and Assisted Heating

**DOI:** 10.3390/polym16131897

**Published:** 2024-07-02

**Authors:** Pedro Rendas, Lígia Figueiredo, Pedro Melo, Carlos Galhano, Catarina Vidal, Bruno A. R. Soares

**Affiliations:** 1 UNIDEMI, Department of Mechanical and Industrial Engineering, NOVA School of Science and Technology, Universidade Nova de Lisboa, 2829-516 Caparica, Portugal; p.rendas@campus.fct.unl.pt (P.R.);; 2 Bioceramed—Cerâmicos para Aplicações Médicas S.A., 2660-360 São Julião do Tojal, Portugal; 3 GEOBIOTEC and Earth Sciences Department, NOVA School of Science & Technology, 2829-516 Caparica, Portugal; 4 Laboratório Associado de Sistemas Inteligentes (LASI), 4800-058 Guimarães, Portugal

**Keywords:** polylactic acid (PLA), polyetheretherketone (PEEK), biopolymers, stationary shoulder friction stir welding (SS-FSW), assisted heating

## Abstract

Additive manufacturing (AM), also known as 3D printing, offers many advantages and, particularly in the medical field, it has stood out for its potential for the manufacture of patient-specific implantable devices. Thus, the unique properties of 3D-printed biocompatible polymers such as Polylactic Acid (PLA) and Polyetheretherketone (PEEK) have made these materials the focus of recent research where new post-processing and joining techniques need to be investigated. This study investigates the weldability of PLA and PEEK 3D-printed plates through stationary shoulder friction stir welding (SS-FSW) with assisted heating. An SS-FSW apparatus was developed to address the challenges of rotating shoulder FSW of thermoplastics, with assisted heating either through the shoulder or through the backing plate, thus minimizing material removal defects in the welds. Successful welds revealed that SS-FSW improves surface quality in both PLA and PEEK welds compared to rotating shoulder tools. Process parameters for PLA welds are investigated using the Taguchi method, emphasizing the importance of lower travel speeds to achieve higher joint efficiencies. In PEEK welds, the heated backing plate proved effective in increasing process heat input and reducing cooldown rates which were associated with higher crystallinity PEEK. Despite these findings, further research is needed to improve the weld strength of SS-FSW with these materials considering aspects like tool design, process stability, and 3D printing parameters. This investigation emphasizes the potential of SS-FSW in the assembly of thermoplastic materials, offering insights into the weldability of additively manufactured biocompatible polymers like PLA and PEEK.

## 1. Introduction

Additive manufacturing (AM), also referred to as three-dimensional (3D) printing, keeps growing in significance for its unique ability to produce unique complex geometries with less material waste in comparison to conventional subtractive manufacturing techniques [1] and for its design flexibility, where 3D-printed parts can be produced based on virtual 3D models generated with Computer-Aided Design (CAD) software [2]. For these reasons, AM has gained the attention of the medical field, where biomaterials compatible with AM techniques can be used in the manufacture of patient-specific medical devices based on medical imaging data. Furthermore, AM of biomaterials is being increasingly studied for the production of intricate 3D scaffold structures for tissue engineering [3,4] and even for the production of Organ-on-Chip models that can mimic tissue environments and improve the assessments of preclinical studies [5,6].

Among potential biomaterials, polymers have stood out for their excellent biocompatibility, wide range of properties, and ability to be processed using Material Extrusion (ME) AM techniques like Fused Filament Fabrication (FFF). Both Polylactic Acid (PLA) and Polyetheretherketone (PEEK) are examples of such materials that have potential applications in the field of orthopaedics for the manufacture of personalized implantable medical devices. PLA is biodegradable and can be used in resorbable components doped with bioactive compounds or bone fixations that do not require surgical removal [7,8,9], while PEEK has a higher strength-to-weight ratio and chemical stability which can be used in long-term load-bearing implant applications making it the leading candidate for the replacement of metallic materials in orthopaedic and trauma treatment applications [10,11]. Additive manufacturing with both these thermoplastic materials has been extensively researched concerning the outcome of mechanical properties and surface quality of 3D-printed components.

Given the dimensional limitations of AM, larger-scale designs require the joining of smaller components using adhesives, mechanical fastening, or welding techniques. Compared to other joining processes, friction stir welding (FSW) could present a better option for the joining of thermoplastic biomaterials since this process does not require the use of additional components or filler material and has a comparatively lower thermal input than most welding techniques [12]. The FSW process was developed by The Welding Institute (TWI) for the solid-state joining of metals and is mostly studied for aluminium alloys [13]. With its development, friction-based processes have been used to incorporate particles in metallic matrices resulting in self-sensing [14] and bioactive materials [15]. For thermoplastics, although FSW technology has been mostly studied and adapted for their welding [16,17], similar developments can be used for the biofunctionalization of these materials like the improvement of antibacterial activity to decrease the risk of infection after implantation [18,19].

Nevertheless, FSW of thermoplastics still challenges researchers due to the difficulty in attaining defect-free joints given the unsteady processing conditions [20]. Unlike the welding of aluminium alloys, FSW of thermoplastics is not an absolute solid-state process given the different molecular weight and lengths of polymeric chains [21]. In addition to this, the lower thermal conductivity typical of these materials hinders the effects of friction and makes the stirring process more difficult. For these reasons, the use of conventional FSW techniques with thermoplastics has produced poor results with the welds displaying voids and root and flash defects together with large amounts of material removal [16,20,22,23]. To address these issues, research has focused on the use of different variations of the FSW technique consisting mostly of heated tools or heated plates [24,25,26,27] and stationary shoulder (SS) tools [28,29]. The stationary shoulder assures that the heat and stirring are generated solely by the rotating pin and allows the shoulder to constrain the stirred material to the joint [30]. Additionally, heated tools can increase process heat input and thus lower the stirred material’s viscosity [31]. This creates a more uniform temperature distribution which can help the flow of material in the stirred region while reducing cooldown rates [32].

Concerning this, the combination of both a stationary shoulder FSW (SS-FSW) tool and assisted heating presents the highest potential for the joining of thermoplastics where the lack of heat generated by the rotating shoulder’s friction can be substituted by an external heating source. This avoids both the material removal and flash defects created by the rotating shoulder while the assisted heating could enhance flow and reduce the void defects on the retreating side. The use of a heated stationary shoulder in the FSW of polymers has been patented [17], and it is often referred to as “hot shoe” FSW. The effects of the “hot shoe” FSW process parameters in the mechanical properties of welded thermoplastics have been investigated [33,34,35], and it is suggested that higher rotational speeds and lower travel speeds produce stronger welds, while the shoulder temperature and axial force could be more dependent on the welded material. Despite the improved weld quality obtained with the “hot shoe” FSW of thermoplastics, more research on the effects of this process’s parameters is required.

Considering research on the FSW of thermoplastics, most studies focus on the use of lower-temperature polymers such as Acrylonitrile butadiene styrene (ABS), polypropylene (PP), Polymethyl methacrylate PMMA, and, mostly, Polyethylene (PE) [31], which have melting temperatures between 115 °C and 200 °C. With these low melting temperatures, FSW has been shown to produce temperatures well above the melting point of the welded material [36]. PEEK is a high-temperature thermoplastic with a melting temperature around 342 °C used in high-performance applications. Also, it is a semi-crystalline polymer like PE, and its mechanical properties are related to its crystalline content. The use of heated FSW tools has been suggested to help increase the crystalline phase of semi-crystalline polymers [37] meaning that under the right conditions SS-FSW has the potential to strengthen the stirred material through the increase in its crystallinity in similarity to the microstructure refinement caused by the FSW of metals [38]. Despite this potential, only a few studies have documented the use of friction-based welding techniques with PEEK [32,39,40], and, to the authors’ knowledge, the joining of PEEK plates using a SS-FSW tool has yet to be studied. The potential of friction stir processing with PEEK has been noticed where interesting studies have been conducted in the dissimilar welding of aluminium alloys to PEEK [41,42,43] and fibre-reinforced PEEK [44,45,46,47], and even to produce a surface PEEK–HA bioactive composite [48,49]. Furthermore, apart from some works on friction-based welding of 3D-printed PLA with rotating shoulder FSW [50] and with rotary friction welding [51], SS-FSW has yet to be tested with 3D-printed plates. Considering the potential of FSW with biomaterials, the use of this technique with additively manufactured components needs to be researched where the processing of biocompatible polymers such as PLA and PEEK becomes more interesting considering their potential patient-specific implant manufacture.

In this work, the weldability of 3D-printed PLA and PEEK plates through FSW is tested using a stationary shoulder tool together with assisted heating provided either by the backing plate or by the tool’s shoulder. Using the developed SS-FSW apparatus, successful welds were obtained for both PLA and PEEK plates from which tensile specimens were cut to test weld strength and joint efficiency. The effects of the tool rotation, travel speed, and external heating temperature on the weld strength of printed PLA were studied using the Taguchi approach. Based on these results, the effects of assisted heating configurations were investigated in their ability to increase process heat input through the weld strength of printed PEEK. In addition to this, calorimetry tests performed on samples from the stirred region of PEEK welds were used to relate the process temperatures to the crystallinity of the samples. Despite promising results obtained with PLA, additional developments are required to increase the joint efficiency of PEEK welds and improve the weldability of high-temperature thermoplastics with SS-FSW.

## 2. Materials and Methods

### 2.1. Materials

For the Design of Experiments (DoE) study of the FSW parameters, 5 mm thick PLA plates of 50 mm width and 47.5 mm length were 3D printed using a Prusa MK3S printer (Prusa Research a.s., Prague, Czech Republic) equipped with a 0.4 mm nozzle. To print these plates, a recycled PLA filament material with a 1.75 mm diameter, tensile strength of 68 MPa, and a tensile elastic modulus of 3.25 GPa was purchased from 3DJake (Niceshops GmbH, Paldau, Austria). The G-code printing files were generated using the slicer software PrusaSlicer (v2.3.3) with 100% rectilinear infill perpendicular to the welding direction. This assures that, after the tensile specimens are cut, the weld is placed at the centre of the specimen while the printed lines are longitudinally aligned. Other print settings include a nozzle temperature of 215 °C, bed temperature of 60 °C, printing speed of 35 $\mathrm{mm}\cdot s^{-1}$, movement speed of 100 $\mathrm{mm}\cdot s^{-1}$, layer height of 0.2 mm, one top and one bottom concentric layers, 2 perimeter lines, and a 50% outline overlap.

For the experiments with PEEK, the plates were additively manufactured using the FFF printer Apium P220 (Apium Additive Technologies GmbH, Karlsruhe, Germany) which was also equipped with a 0.4 mm nozzle and includes an adaptive heating system, referred to as zone heater, that allows for high-quality PEEK prints. A neat PEEK filament (Apium PEEK 450 Natural, Apium Additive Technologies GmbH, Karlsruhe, Germany) with a diameter of 1.75 mm was used to print the plates which was previously dried in the Apium F300 filament dryer for 4 h at 120 °C and then maintained at 60 °C for conditioning and printing. In this case, the G-code printing files were generated using the slicer software Simplify3D (v4.1.2) with the default printing temperatures for PEEK of 485 °C for the nozzle, 130 °C for the build plate, and 130 °C for the zone heater. Deposition parameters like layer height, infill, and build orientation are equivalent to the ones used in the PLA plates while printing speed and movement speed were adjusted to 2000 $\mathrm{mm}\cdot s^{-1}$ and 4800 $\mathrm{mm}\cdot s^{-1},$ respectively. Additionally, all PEEK plates were printed with a large brim width of 14 mm to minimize warping defects.

### 2.2. FSW Apparatus

**Figure 1:**
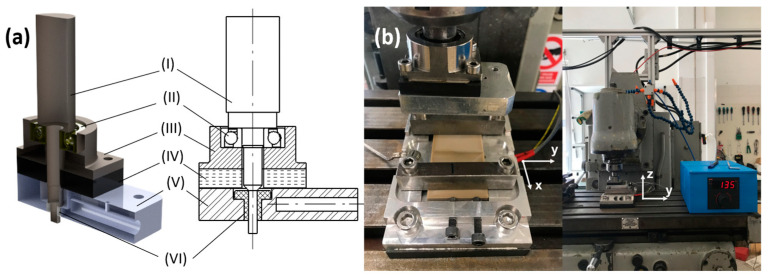
Stationary shoulder FSW tool’s assembly (**a**) with rotating pin shaft (I), angular contact bearing (II), bearing housing (III), thermal insulating spacer (IV), aluminium shoulder (V), and low-friction sleeve (VI) and conventional milling machine setup (**b**).

The SS-FSW tool and the heated fixture for the plates were both designed and manufactured in-house. The SS-FSW tool is an assembly of six main components (Figure 1a) consisting of an aluminium shoulder, a bakelite thermal insulating layer, a low friction sleeve, an angular contact bearing, a bearing housing component, and a rotating pin tool. Among other design considerations, the PTFE material was chosen for the sleeve for its remarkably low friction coefficient and temperature resistance. The aluminium shoulder included a hole along its longitudinal axis for the resistance cartridge heater so that heat could be provided through the shoulder as a “hot shoe” configuration. A type K thermocouple was fastened to the top surface of the shoulder to measure an equivalent temperature to the bottom surface of the shoulder. With this, the assembled tool’s main dimensions correspond to a shoulder width of 45.6 mm, shoulder length of 74 mm, pin diameter of 5 mm, and a pin length of 4.3 mm. For the pin geometry, a cylindrical left-hand threaded pin was selected based on trial tests performed in PLA plates with conventional FSW tools.

In addition, a fixture component was also machined out of aluminium where the plates were fastened to perform the welds. This backing plate fixture includes two lateral screws that put pressure on the joint’s surfaces and a slot underneath for a plate resistance heater. Similarly, the thermocouple was fastened to the same surface of the fixture that is in contact with the PEEK plates to obtain more accurate temperature reads. The welds were performed using a conventional 3-axis milling machine and the assembly of the PEEK plates into the heated fixture together with the SS-FSW tool can be seen in Figure 1b.

### 2.3. Experimental Procedure

Square butt welds were performed in 50 mm × 47.5 mm plates with 5 mm of thickness. The plates were secured to the milling machine’s table using the designed aluminium backing plate fixture, and the butt weld was performed along the 50 mm side of the plates aligned with the Y-axis. The pressure bars that secure the plates in place were tightened after the travel screws to ensure pressure on the joint’s surfaces.

All the welds were performed with position control for plunge depth. For this, the tool pin was aligned with the weld, and the plunge depth was measured from a Z-axis zero set in the feed handwheel with the pin touching the top surface of the plates. A pin plunge depth of 4.8 mm was chosen according to trial runs performed in PLA plates. This depth is 0.5 mm greater than the length of the pin thus compressing the plates and helping keep axial pressure in the weld region. This may help compensate for the limitations of position-controlled FSW compared to force-controlled FSW since axial force control has been shown to help steady the conditions of “hot shoe” FSW and produce stronger welds [52]. In addition to this, all welds were performed with dwell times of 10 s after plunge and before retraction to create more uniform temperature profiles concerning the heat provided by the external heat source and the friction of the rotating pin. Although this parameter has been addressed mostly for the friction stir spot welding of polymers [53,54,55], longer dwell times have been reported to increase the heat input of FSW performed on aluminium alloys [56]. Lastly, a thermal imaging infrared (IR) camera Fluke Ti400 (Fluke Corporation, Everett, WA, USA) was used to monitor both the plates and the tool’s temperatures during and after the FSW process.

With the plates welded, tensile specimens were cut using a HAAS Mini Mill CNC equipped with a 6 mm contour mill tool. The tensile testing was performed according to the ISO 527 specifications using a universal testing machine (MTS 312.31, MTS Systems Corp., Eden Prairie, MN, USA) with a 100 KN load cell and constant crosshead speed of 0.01 mm/s. For this, the tensile specimens were cut following the dimensions specified for type 1BA with an overall length of 95 mm and 5 mm of thickness corresponding to the welded plates length and thickness. Test specimens were also obtained from unwelded printed plates of 30 mm × 95 mm × 5 mm plates to obtain the strength of the base material used in tensile joint efficiency measurement. Following what was performed in other works [36,57], joint efficiency was obtained as the ratio between the strength of the welded specimen and the strength of the base material. Lastly, the outer section remaining from the cut of the tensile specimens was used to observe the welds’ cross section and to take samples of the weld nugget for differential scanning calorimetry (DSC) analysis.

### 2.4. SS-FSW Parameters and Assisted Heating

Concerning FSW parameter adjustment, the DoE study was conducted to determine optimal combination of rotational speed (RS), travel speed (TS), and backing plate temperature (BPT) using the Taguchi method with the L9 orthogonal array, as detailed in Table 1. The parameters used in the experiments, despite being selected to minimize this issue, do not vary linearly since the conventional milling machine used to perform the welds only allows for the selection of discrete values of rotational speed and travel speed. For each parameter combination, a minimum of 3 tensile specimens were obtained from individual welds performed in PLA plates and tested for their tensile strength and corresponding relative joint efficiency. Given the difficulty in maintaining weld conditions through the experiments and the frailty of the welds produced with some of the parameter combinations, some welds were repeated and tested to substitute outliers. With the results from the experiments, Analysis of Variance (ANOVA) was used to assess each parameters contributions and respective significance. This analysis was performed using the Minitab statistical analysis software (version 19.2020.1).

Following the results from the Taguchi analysis on the SS-FSW of PLA plates, both RS and TS were fixed to test the effects of different assisted heating temperatures in the weldability of PEEK printed plates. In similarity to the temperatures tested with PLA, the experiments’ temperatures were chosen around PEEK’s glass transition temperature of 143 °C [58]. Considering this, SS-FSW was used to perform welds on 3D-printed PEEK plates with no assisted heating (ambient temperature of 28 °C) and with assisted heating at the temperatures of 125 °C, 145 °C, and 165 °C. Tensile test specimens were cut from welded plates and tested to investigate the effects of assisted heating temperature in the tensile strength and corresponding joint efficiency of the welds.

### 2.5. Thermal Analysis

DSC analysis was carried out in samples taken from the nugget of the weld to assess the effects of the process temperatures in the crystallization of PEEK. For this, DSC tests were conducted using a NETZSCH DSC 204 F1 Phoenix calorimeter (Erich NETZSCH GmbH & Co. Holding KG, Selb, Germany) and the thermal analysis was performed using the NETZSCH Proteus software. For this, PEEK weld samples were tested with heating/cooling rates of 10 °C/min and a maximum temperature of 490 °C for 5 min. With these tests, it was possible to obtain other thermal properties of the samples such as the glass transition, melt, and crystallization temperatures and, most importantly, the crystalline phase percentage for the tested samples. To determine this crystallinity percentage, the ratio between the heat required to melt the sample and the theoretical heat of fusion of fully crystalline PEEK was calculated following the same method used in other works [59,60,61]. This way, the percentage of the crystalline phase for FSW-processed PEEK was determined with the following equation:

(1) $${Χ}_{c}\;\left(\%\right)=\frac{\Delta H_{endo}-\Delta H_{exo}}{\Delta H_{c}}$$

where $\Delta H_{endo}$ is the melt enthalpy measured as the area under the melting endothermic peaks, $\Delta H_{exo}$ is the “cold” crystallization enthalpy measured as the absolute value of the area above the exothermic peaks of the heating curve, and $\Delta H_{c}$ is the theoretical melt enthalpy of fully crystalline PEEK. According to the crystallinity percentage determination methods used by other authors, this theoretical melt enthalpy of fully crystalline PEEK corresponds to $130\;J\cdot g^{-1}$ [62].

## 3. Results and Discussion

### 3.1. FSW Parameters on the Relative Weld Efficiency of 3D-Printed PLA

**Figure 2:**
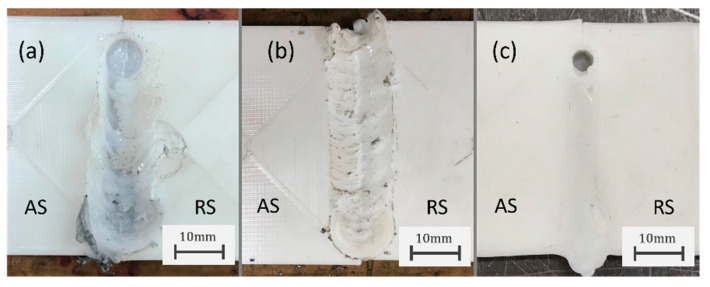
PLA joints produced on trial runs (900 rev·min^−1^, 11 $\mathrm{mm}\cdot \min^{-1}$) with a rotating shoulder FSW tool with shoulder plunge (**a**), with clockwise rotation on left-hand threaded pin (push-down) (**b**), and with stationary shoulder FSW tool (**c**); (AS-Advancing side; RS-Retreating side).

A series of trial runs were performed using 3D-printed PLA plates to test the tools and to choose tool geometry and parameters. The first trials were performed using rotating shoulder FSW tools used for aluminium alloys. For this, a concave shoulder with a 14 mm diameter and a left-hand threaded pin with a 5 mm diameter and a 4 mm length produced the best-looking welds among the available configurations for the FSW tool. The use of these rotating shoulder FSW tools resulted in large amounts of material removal and flash defects (Figure 2a). This is one of the reasons why research has focused on the use of SS-FSW for thermoplastics since this technique allows the pin to stir the material while the shoulder contains it in the joint [30].

In addition to this, the use of clockwise rotation with the left-hand threaded pin creates a push-down effect [63] which helps keep the stirred material in the joint and reduces flash defects [64]. Despite this, considering the low thermal conductivity of thermoplastic materials like PLA, the heat generated from shoulder friction appears insufficient for the weld region to reach the necessary visco-plasticity to be stirred by the pin and thus resulted in unsuccessful welds. Nevertheless, the use of the push-down effect proved efficient for the reduction in flash defects (Figure 2b) and, for this reason, the stationary shoulder tool used in this work features a left-hand threaded pin which will be rotated clockwise. This effect, in addition to the heat provided by the assisted heating should help reduce material removal and increase joint efficiency.

With this tool configuration, another set of trial runs were conducted where significant improvements were seen in the surface quality of the weld even without external heating (Figure 2c). The implementation of 10 s dwell times after plunge and before pin retraction together with the 0.5 mm of plate compression, as described in the experimental procedure section, produced steadier conditions where repeatability between trials was easier to achieve. This addresses the consistency issues reported by Lambiase et al. [20] and can support the use of SS-FSW for the joining of thermoplastics. Furthermore, the welds produced with the developed tool showed little evidence of the previously reported flash defects. With metallic materials, the use of rotating shoulder FSW tools is required since the rotating pin affects mostly the thermo-mechanically affected zone while the rotating shoulder’s friction contributes to expand the heat affected zone. However, given the low thermal conductivity of polymers, the friction from the shoulder generates heat mostly on the plates’ surface which is insufficient to lower the stirred material’s viscosity. With SS-FSW, the flow of material in the stirred region is created by the pin while the shoulder contains the material in the joint [30].

With the assisted heating stationary shoulder tool, different FSW parameters were tested according to the combinations presented in Table 1. The results for tensile strength of the welded and base material specimens are provided in Table 2 together with the relative joint efficiency calculated for each parameter combination. As a consequence of 3D printing deposition and specimen cutting, the average tensile strength of 40.7 MPa obtained for the base material is about 60% of the tensile strength reported for the PLA filament material. Considering this, and despite the low tensile strength measured in welded specimens, some parameter combinations resulted in relatively high joint efficiencies. The highest strength result among all tensile tests of 38.2 MPa was obtained with combination C7 (1400 rev·min^−1^, 11 mm·min^−1^, and 60 °C) corresponding to a joint efficiency of 93.8%. However, considering the difficulty in achieving repeatability in weld conditions, the highest average strength, still obtained with C7, corresponds to a lower 30.8 MPa and a joint efficiency of 75.6%. These results for highest strength are considerably higher than those reported by Senthil and Kumar [57] and are similar to the results presented by Vidakis et al. [50], both regarding the joining of 3D-printed PLA sheets with rotating shoulder FSW.

**Figure 3:**
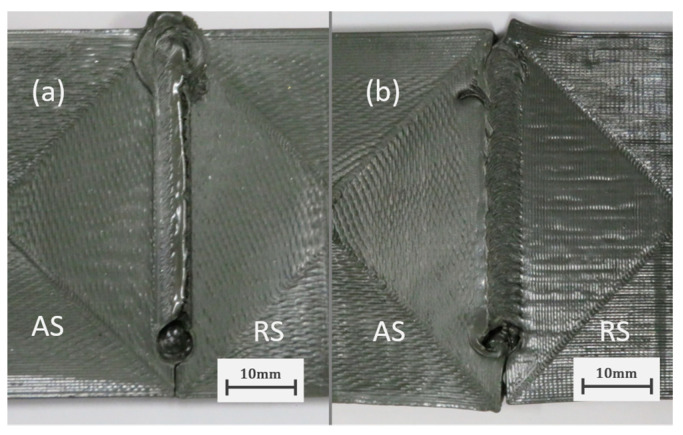
Surface quality of welds produced with parameter combinations C (**a**) and C7 (**b**); (AS-Advancing side; RS-Retreating side).

Among the tested parameters, an inverse relation between travel speed (TS) and the strength of the welded specimens was noticed. The three lowest average tensile strengths were obtained with parameters C3, C6, and C9, all of which use a maximum TS of 28 mm·min^−1^. In the plates welded with these parameters, there is evidence of a large-scale void defect forming along the retreating side of the weld that cannot be seen in the higher efficiency welded plates like C7 (Figure 3). This type of defect has been attributed to the insufficient heat input of high TS which, considering the low thermal conductivity, is unable to make the material reach the necessary viscosity for it to be stirred [35,52]. The appearance of these defects seems to be in line with demonstrations of Zhang et al. [65] where the torsion and circumventing velocity have different directions in the retreating side, thus hindering the stirring effects of the pin. Considering this, lower travel speeds can improve the flow of material through the increase in heat input and stirring effects. The importance of TS was also reported in previous research in the FSW of 3D-printed PLA plates where the strongest welds were produced with even lower speeds of 6 mm·min^−1^ [50].

**Figure 4:**
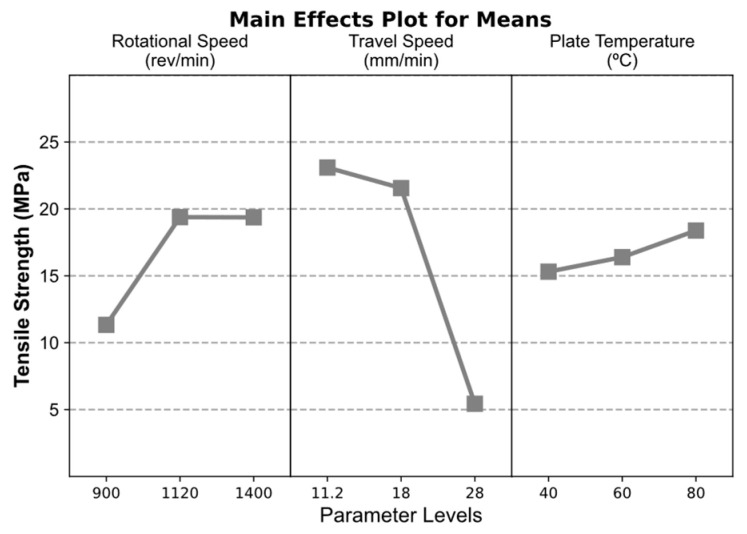
Taguchi analysis main effects plot for tensile strength means of welded PLA specimens.

Apart from this, the effects of RS and BPT parameters are less evident in the surface quality of the welds and in the tensile test results. The results for the main effects from the Taguchi analysis are presented in Figure 4 where the main effects for means was considered in order to identify the parameters effects on the average joint efficiency of FSW welds. According to this analysis, the optimal combination of FSW parameters to maximize average tensile strength, and consequently joint efficiency, consists of intermediate to high RS of 1120 to 1400 rev·min^−1^, low TS of 11.2 mm·min^−1^, and high BPT of 80 °C. As identified in the results presented in Table 2, the high TS of 28 mm·min^−1^ resulted in the lowest plotted effect on mean tensile strength. These results suggest that maximizing process heat input by increasing RS and BPT while decreasing TS can improve joint efficiency in the SS-FSW of thermoplastic materials.

Following these results, an ANOVA was conducted to assess the statistical significance of differences between the means with different parameters and their contribution to the mean tensile strength of the welds. The results from this analysis are provided in Table 3. From the tested parameters, both RS and TS display statistical significance (p<0.05) while BPT is not considered statistically significant with a considerably high *p-*value. Concerning each parameters contribution, TS has the highest impact in the strength of the welds with a contribution of 57% while considerably lower contributions are attributed to RS and BPT of 12.8% and 1.4%, respectively. These results, together with the results from the Taguchi analysis, suggest that, in addition to the increase in heat input with SS-FSW parametrization, the strength of the welds is mostly affected by travel speed where lower speeds improve stirring conditions and reduce the presence and size of void defects on the retreating side of the weld.

**Figure 5:**
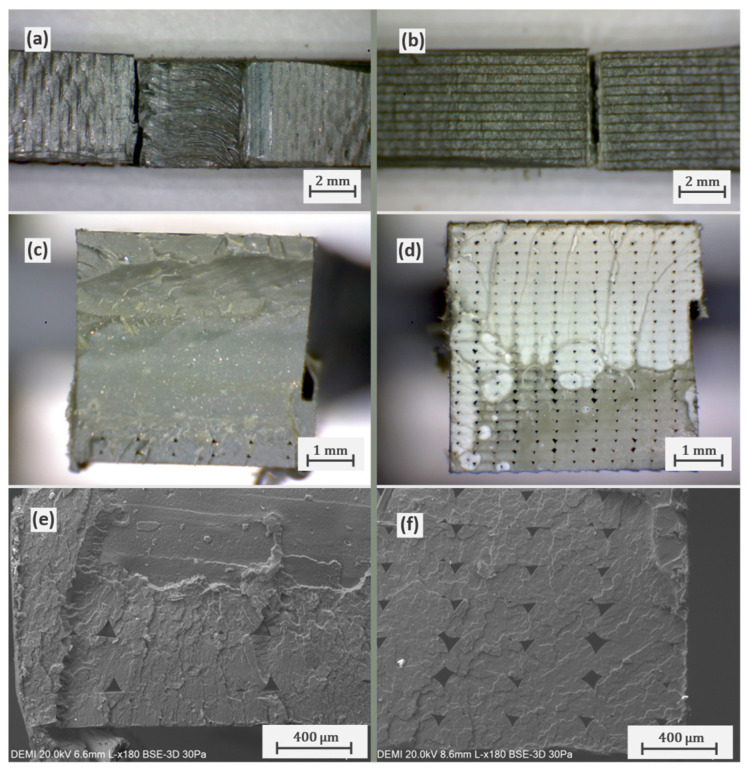
Fracture surface comparison between welded (C7) and unwelded PLA test specimens in top view (**a**,**b**), fracture surface photographs (**c**,**d**), and SEM images (**e**,**f**).

Even with little evidence of retreating side void defects in the higher joint efficiency welds like C7, all the tested specimens fractured in the interface between the base material and the nugget region on the retreating side. Figure 5 shows the comparison between the fracture surface of the highest efficiency weld performed with parameters C7 and the fracture surface of the base material tensile specimen. In the welded specimen, fracture on the retreating side (Figure 5a) occurred mostly in the stirred region with the exception of the bottom section of the surface which fractured through infill lines of the 3D-printed plates (Figure 5c). SEM images of this region (Figure 5e) show a rough surface and evidence of void defects created through 3D printing deposition closely resembling the fracture surface of the base material tensile specimens (Figure 5d,f). This suggests that the bottom section of the weld was subject to more favourable bonding conditions which can be attributed to higher temperatures near the backing plate or to the pushdown effect from pin stirring.

These results indicate that 3D-printed PLA can be successfully welded using SS-FSW with assisted heating. However, the strength of the weld is closely linked to the processing conditions determined by FSW parameters. This study reveals that a low travel speed is necessary for weldability, as it reduces large-scale void defects on the retreating side and allows the stirred material to fill the entire weld region. This means that FSW of thermoplastics has significantly lower completion rates than the FSW of aluminium alloys. Apart from this, higher rotation speed and backing plate temperature can enhance material flow by increasing heat input and thus compensate for the lack of shoulder friction. Nevertheless, these parameters were found to be of less significance, perhaps because of the low transition temperatures of PLA, which do not require considerable heat input in the process. Despite the challenges with process stability, there were two welds obtained in this work that surpassed 90% of joint efficiency suggesting that, with adequate process parametrization, SS-FSW can produce high-performance welds in 3D-printed thermoplastics. Nevertheless, further investigations on the use of different pin geometries and assisted heating configurations are required for the selection of the adequate FSW process variation for the joining of thermoplastic materials.

### 3.2. Assisted Heating SS-FSW of 3D-Printed PEEK

**Figure 6:**
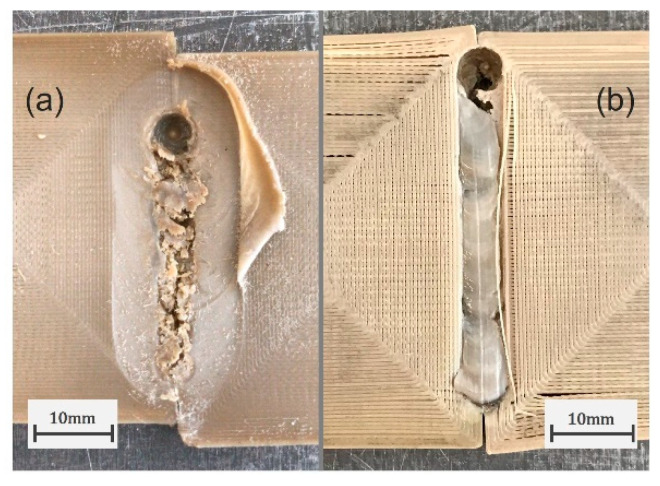
Surface quality of PEEK welds produced with 145 °C of backing plate temperature, 1400 RPM and 11 $\mathrm{mm}\cdot \min^{-1}$ using a rotating shoulder FSW tool (**a**) and the developed SS-FSW tool (**b**).

Following the conclusions taken from the experiments performed using PLA plates, the same SS-FSW process configuration was used to assess the weldability of 3D-printed PEEK. Similar to what was seen with PLA in Figure 2, the use of the SS-FSW technique proved essential for the joining of PEEK since the use of a rotating shoulder tool at 1400 rev/min, 11.2 $\mathrm{mm}\cdot \min^{-1}$, and 145 °C of backing plate temperature was unable to join the printed PEEK plates while the SS-FSW tool produced a seemingly solid joint (Figure 6). Compared to PLA, PEEK has a much higher glass transition and melt temperature meaning that the heat input obtained from mechanical stirring is less effective. With high-temperature materials like PEEK, the assisted heating of the welded plates becomes more relevant since it can help increase process heat input and thus improve material flow. Considering this, based on the experiments conducted with PLA and some trial runs with PEEK, the rotational speed of 1400 RPM and the travel speed of 11.2 $\mathrm{mm}\cdot \min^{-1}$ obtained from the Taguchi analysis with PLA were chosen to investigate the effects of the assisted heating temperatures in the SS-FSW of PEEK. For this, the heated backing plate was selected as the main configuration given the semi-crystalline nature of PEEK since it allows for a more uniform heating of the plates and can remain active after removal of the FSW tool. This allows for a slower cooling of the weld and could ultimately create stronger weld regions.

Despite its results, this study does have some limitations considering challenges identified for the SS-FSW of PEEK. With this material, steady-state conditions were even more difficult to obtain which hindered repeatability throughout the welding experiments. Another critical issue was with the tool’s durability. The higher loads and temperatures derived of PEEK’s FSW caused tool overheating. These issues were intensified with assisted heating through the shoulder despite the thermal insulation between the tool’s bearing housing and the heating element. Due to this, only the maximum temperature of 165 °C was tested using the heated shoulder configuration. Furthermore, PEEK plates’ 3D printing quality using the printer’s default parameters also resulted in low adhesion prints where voids formed between deposited lines. Further developments in the tool’s design and in the 3D printing of PEEK plates are required to further investigate the weldability of PEEK using this technique.

**Figure 7:**
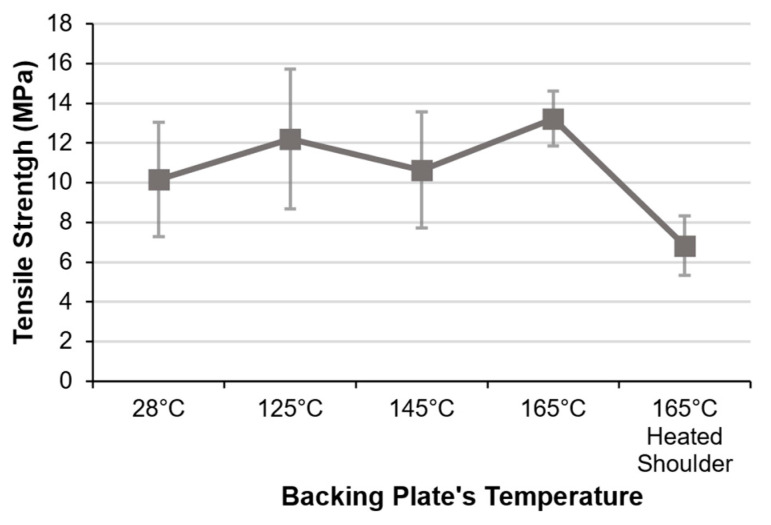
Tensile strength of welded PEEK specimens obtained with different assisted heating temperatures.

**Figure 8:**
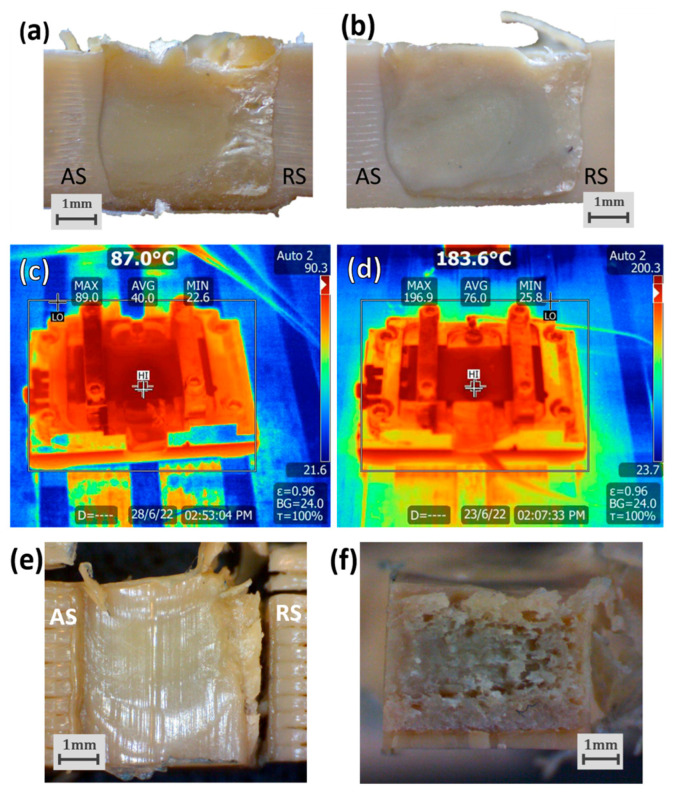
Nugget cross-section of PEEK welds produced with no assisted heating (**a**) and with a backing plate temperature of 165 °C (**b**); IR thermal images displaying the temperatures of the weld regions after tool retraction for 165 °C of shoulder temperature (**c**) and 165 °C of backing plate temperature (**d**); Fracture placement (**e**) and surface morphology (**f**) of tensile specimen produced with 145 °C of backing plate temperature; (AS-Advancing side; RS-Retreating side).

Notwithstanding the mentioned challenges, SS-FSW of PEEK printed plates was performed for different temperature conditions and tested for weld strength. The tested temperatures for the backing plate were chosen according to PEEK’s glass transition temperature of 143 °C [58], and, with this, welds were performed either without a heated plate (at an ambient temperature of about 28 °C) or with the plate heated to 125 °C, 145 °C, and 165 °C. The results for the specimens’ average tensile strength are plotted in Figure 7. The highest strength of 13.2 MPa was obtained with the maximum tested temperature of 165 °C; however, these results may be of little statistical significance given the large variances observed with the tensile tests. Still, the use of higher temperatures seems to increase the heat input and lower PEEK’s viscosity which results in more uniform weld sections. This also increases the effects of the pin stirring induced flow and seems to decrease the appearance of void defects on the weld’s retreating side as seen in the nugget’s microscopic photographs (Figure 8a,b). In these micrographs, it is also possible to observe the different colour tones in different nugget regions. These tones have been associated with PEEK’s crystalline (light beige) and amorphous (dark brown) phases [66] and thus can also be related to PEEK’s cooldown rates. Considering this, the darker region at the root of the weld performed with no backing plate heating can correspond to a more amorphous PEEK region (Figure 8a). Furthermore, dark contour regions can be seen around the sections of both nuggets in their respective images. This amorphous contour region can be associated with polymer chain damage caused by high shear in the transition between the stirred region and the base material.

Since higher crystallinity PEEK displays higher strength and rigidity [67], there is also the possibility to improve the mechanical properties of the nugget region using heated backing plates which increases its crystallinity by slowing cooling rates [68]. For this reason, a shoulder temperature of 165 °C was tested to compare its effects to the heated backing plate. The tensile strength result for the welds obtained with the heated shoulder is also plotted in Figure 7 along with the results for the heated backing plate. The average strength of 8.2 MPA displayed for the heated shoulder specimens was lower than any displayed for the heated backing plate specimens. The advantage of the heated backing plate against the heated shoulder has been previously reported for other semi-crystalline polymers such as PE [24], where the heated plate can heat the plates before, during, and after the welding process. This can also be seen in thermal imaging taken with the IR camera right after tool retraction where the heated backing plate was able to heat the PEEK plates more efficiently and maintain them at high temperatures after the weld (Figure 8c,d).

The heated backing plate allows for more controlled heating and cooling rates that can reduce thermal stresses in the joint and can be especially relevant for semi-crystalline materials like PEEK. Nevertheless, achieving this controlled cooling presents additional challenges. The heating of the SS-FSW tool closer to processing temperatures for materials can injure the tool’s durability and may require more developments such as incorporated cooling systems that keep the bearing at lower temperatures. Furthermore, the higher crystalline PEEK resulting from lower cooling rates also displays lower fracture toughness [67] which can result in weaker welds. For these reasons, future developments could explore the relations between different cooling conditions and post-processing heat treatments in the crystallinity and strength of welded PEEK plates.

Apart from the tensile test results, samples were taken from the central section of the nugget for each tested temperature and submitted to DSC analysis. This allowed for the assessment of whether the heated backing plate produces higher crystallinity weld sections compared to the heated shoulder and, if so, which temperature produces the best results. The DSC results for the glass and melt transitions taken from the heating curve as well as the results for the “hot” crystallization taken from the cooling curve are provided in Table 4. These results refer to samples taken from welds produced at ambient temperature, with a heated backing plate and with a heated shoulder.

Observing the DSC heating curves from the tested samples, no exothermic “cold” crystallization peak was found apart from a very slight peak found for the heated shoulder sample with its peak at around 175 °C. This already suggests that the samples obtained had a relatively high crystallinity percentage since no further significant crystallization was observed in the heating of the samples. Focusing on the crystallinity percentages obtained from the DSC results, the highest crystallinity percentage was obtained with a backing plate temperature of 125 °C which was the lowest temperature tested for this configuration.

The heated backing plate resulted in weld regions with slightly higher crystallinity compared to the ambient temperature weld. More significantly, the heated shoulder configuration resulted in a weld region with a noticeably lower crystallinity which justifies the small exothermic peak described above. This can be explained by the faster cooldown observed in the weld, consequent of the heated shoulder’s retraction after the weld. These results, following the results from the tensile tests, further support the use of heated backing plates instead of a heated shoulder for the SS-FSW of high temperature thermoplastics such as PEEK. Despite this, crystallinity measurements taken from DSC tests lack precision due to a variety of factors like sample preparation and enthalpy calculation. Considering this, further research on the crystallinity of FSW-processed PEEK is required.

The use of the developed SS-FSW tool produced interesting results in the joining of both 3D-printed PLA plates and 3D-printed PEEK plates. Significant improvements in weld quality can be seen for both PLA and PEEK using the developed stationary shoulder tool. Additionally, reducing travel speed helps reduce the presence and size of large-scale void defects forming on the retreating side of the weld and thus improves weld strength. For high-temperature thermoplastics like PEEK, providing heat through the backing plate can help increase the heat input of the process which can help material flow while controlling cooldown rates and promoting PEEK’s crystallization.

Despite these improvements, this work’s results for the FSW of PEEK plates displays some shortcomings. Disregarding the increases observed for higher plate temperatures, the tensile strengths obtained for the PEEK tensile specimens were considerably low. As a high-performance thermoplastic, PEEK displays high tensile strength compared to other materials, and the tensile specimens cut from printed plates to represent the base material displayed an average tensile strength of 56.3 MPa. This strength is much lower than that reported in research for 3D-printed PEEK, which can be as high as 98.9 MPa [69]. This suggests that the default printing parameters used to print the plates produced low-performance PEEK samples with poor interfacial adhesion between the lines and layers of the 3D print. The lack of coherence from the printed material transitions directly to the solid nugget region (Figure 8e) which, in combination with the void defects formed by the weld, creates a weak interface in the retreating side where fracture occurs (Figure 8f).

Nevertheless, the SS-FSW of 3D-printed PEEK plates produced average joint efficiencies for tensile strength between 18.1% and 23.5% with the highest value obtained with the plate temperature of 165 °C. These results for weld strength and joint efficiency, despite being considerably low, are higher than those reported by Jose and Panneerselvam [40], which to our knowledge is the only other work addressing welding of PEEK using FSW techniques. With this, it seems that the use of stationary shoulder tools together with a heated backing plate produces the best results in the weldability of PEEK plates. Nevertheless, further research in the development of SS-FSW tools and procedures for the joining of high-temperature thermoplastics such as PEEK is required to improve the joint efficiency and performance of these welds.

## 4. Conclusions

Stationary shoulder FSW with assisted heating was tested for the joining of 3D-printed PLA and PEEK plates. Compared to rotating shoulder tools, SS-FSW produced noticeable improvements in the surface quality of welds performed in both PLA and PEEK. Based on the results and discussion, the conclusions of this work’s investigation on the SS-FSW of 3D-printed biocompatible thermoplastics can be summarized as follows:

- The use of stationary shoulder tools for the FSW of thermoplastics is more effective in containing the material in the stirred region and reduces material removal and flash defects observed with rotating shoulder tools;
- The lowest tested travel speed of 11.2 $\mathrm{mm}\cdot \min^{-1}$ reduced the presence and size of void defects appearing on the retreating side and resulted in the highest joint efficiencies;

- The parameter combination that maximizes the average strength of the weld corresponds to intermediate–high RS of 1120–1400 rev·min^−1^, low TS of 11.2 mm·min^−1^, and high BPT of 80 °C. A significant contribution of about 58% is attributed to travel speed while backing plate temperature was not statistically significant in the strength of the welds;

- The highest strength of 38.2 MPa corresponding to a joint efficiency of 93.8% was obtained with 1400 rev·min^−1^, 11.2 mm·min^−1^, and 80 °C which is higher than the results obtained in previous research. Nevertheless, these parameters resulted in an average tensile strength of 30.8 MPa and joint efficiency of 75.6%;

- The higher heat input required for the SS-FSW of high-temperature thermoplastics such as PEEK can be provided through assisted heating. This can improve material flow in the stirred region since the highest tested backing plate temperature of 165 °C was found to reduce void defects appearing in the retreating side;
- The use of a heated backing plate is a more effective configuration for the assisted heating compared to the heated shoulder in the “hot shoe” configuration since it allows for a more controlled cooling of the weld and thus can promote crystallization of FSW-processed PEEK.

Still, despite the improvements in the weldability of these materials with assisted heating SS-FSW, there are still some shortcomings that need to be addressed in future research to enhance weld quality and strength. Process stability can be improved by including axial force control while different tool configurations need to be tested, namely different pin geometries and sizes. For PEEK, tool design needs to be improved considering the higher operating loads and temperatures associated with this material. In addition to this, 3D printing parametrization can also be addressed where different deposition patterns can influence the transition from the base material to the stirred region and therefore can be related to weld strength. This can be especially relevant for the weldability of 3D-printed PEEK plates where the low weld strength corresponding to joint efficiencies of only 18.1–23.5% can be improved. With further developments, the use of SS-FSW can become viable for the assembly of thermoplastic materials while opening opportunities for the investigation of friction stir processing applied to high-performance 3D-printed thermoplastics such as PEEK.

## Figures and Tables

**Table 1:** Taguchi array L9 with the FSW parameter combinations used in experiments.

	RS (rev/min)	TS (mm/min)	BPT (°C)
C1	900	11.2	40
C2	900	18.0	60
C3	900	28.0	80
C4	1120	11.2	80
C5	1120	18.0	40
C6	1120	28.0	60
C7	1400	11.2	60
C8	1400	18.0	80
C9	1400	28.0	40

**Table 2:** Welded PLA specimens tensile test results and calculated joint efficiency.

	Tensile Strength (MPa)	Joint Efficiency (%)
Base Material	40.7 ± 1.5	—
C1	11.7 ± 1.9	28.6 ± 4.7
C2	19.2 ± 3.2	47.2 ± 7.8
C3	3.1 ± 3.8	7.7 ± 9.4
C4	26.9 ± 2.8	66.0 ± 6.9
C5	21.2 ± 1.3	52.2 ± 3.3
C6	10.1 ± 2.1	24.7 ± 5.1
C7	30.8 ± 11.9	75.6 ± 29.2
C8	24.22 ± 9.4	59.5 ± 23.2
C9	3.12 ± 2.4	7.7 ± 5.9

**Table 3:** Analysis of Variance table for tensile strength of welded PLA specimens.

	Degrees of Freedom	Sum of Squares	Mean Square	F-Value	*p*-Value	Contribution (%)
RT	2	388.44	194.22	4.47	0.025	12.8
TS	2	1722.47	861.23	19.81	0.000	57.0
BPT	2	43.76	21.88	0.50	0.612	1.4
Error	20	869.56	43.48			28.8
Total	26	3024.23				100.0

**Table 4:** DSC results for thermal properties and crystallinity percentage; (AT-Ambient temperature, BP-Backing Plate; HS-Heated Shoulder).

	$T_{g}\;\left({}^{\circ}C\right)$	$T_{hC\;}\left({}^{\circ}C\right)$	$∆H_{hC}\;\left(J.g^{-1}\right)$	$T_{m}\;\left({}^{\circ}C\right)$	$∆H_{m}\;\left(J.g^{-1}\right)$	${Χ}_{c}\;\left(\%\right)$
AT 28 °C	149.7	293.8	−33.68	344.3	36.24	27.9%
BP 125 °C	152.7	294.8	−44.04	345.5	40.57	31.2%
BP 145 °C	148.8	294.5	−39.34	347.9	36.86	28.4%
BP 165 °C	145.7	294.9	−42.68	344.8	37.34	28.7%
HS 165 °C	144.7	294.4	−45.90	347.6	31.02	23.9%

## Data Availability

Data are contained within the article.

## References

[1] Sachs E., Cima M., Cornie J., Brancazio D., Bredt J., Curodeau A., Fan T., Khanuja S., Lauder A., Lee J. (1993). Three-Dimensional Printing: The Physics and Implications of Additive Manufacturing. CIRP Ann. Manuf. Technol..

[2] Gibson I., Rosen D., Stucker B., Khorasani M. (2021). Additive Manufacturing Technologies.

[3] Haryńska A., Carayon I., Kosmela P., Szeliski K., Łapiński M., Pokrywczyńska M., Kucińska-Lipka J., Janik H. (2020). A Comprehensive Evaluation of Flexible FDM/FFF 3D Printing Filament as a Potential Material in Medical Application. Eur. Polym. J..

[4] Garcia J., Yang Z.L., Mongrain R., Leask R.L., Lachapelle K. (2018). 3D Printing Materials and Their Use in Medical Education: A Review of Current Technology and Trends for the Future. BMJ Simul. Technol. Enhanc. Learn..

[5] Sadeghzade S., Hooshiar M.H., Akbari H., Tajer M.H.M., Sahneh K.K., Ziaei S.Y., Jalali F., Akouchakian E. (2024). Recent Advances in Organ-on-a-Chip Models: How Precision Engineering Integrates Cutting Edge Technologies in Fabrication and Characterization.

[6] Galateanu B., Hudita A., Biru E.I., Iovu H., Zaharia C., Simsensohn E., Costache M., Petca R.C., Jinga V. (2022). Applications of Polymers for Organ-on-Chip Technology in Urology. Polymers.

[7] Middleton J.C., Tipton A.J. (2000). Synthetic Biodegradable Polymers as Orthopedic Devices. Biomaterials.

[8] Athanasiou K.A., Niederauer G.G., Agrawal C.M. (1996). Biocompatibility and Clinical Applications of Polylactic Acid/Polyglycolic Acid Copolymers. Biomaterials.

[9] Destefano V., Khan S., Tabada A. (2020). Applications of PLA in Modern Medicine. Eng. Regen..

[10] Kurtz S.M. (2012). An Overview of PEEK Biomaterials. PEEK Biomaterials Handbook.

[11] Green S., Schlegel J. (2001). A Polyaryletherketone Biomaterial for Use in Medical Implant Applications. Polym. Med. Ind. Proc. Conf. Held Bruss..

[12] Hussain R.K., Majeed A.A. (2018). Thermoplastics Polymers Friction Stir Welding: Review. Int. J. Eng. Technol..

[13] Thomas W.M., Nicholas E.D., Needham J.C., Murch M.G., Temple-smith P., Dawes C.J. (1991). Friction Stir Butt Welding. G.B. Patent Application.

[14] Ferreira P.M., Machado M.A., Carvalho M.S., Vidal C. (2023). Granting Sensorial Properties to Metal Parts through Friction Stir Processing. Meas. J. Int. Meas. Confed..

[15] Vidal C., Alves P., Alves M.M., Jo M., Fernandes H., Grenho L., In P.L., Ferreira F.B., Santos T.G., Santos C. (2022). Fabrication of a Biodegradable and Cytocompatible Magnesium/Nanohydroxyapatite/Fluorapatite Composite by Upward Friction Stir Processing for Biomedical Applications. J. Mech. Behav. Biomed. Mater..

[16] Arici A., Sinmaz T. (2005). Effects of Double Passes of the Tool on Friction Stir Welding of Polyethylene. J. Mater. Sci..

[17] Nelson T.W., Sorenson C.D., Johns C.J. (2004). Friction Stir Welding of Polymeric Materials 2004. U.S. Patent.

[18] Ishihama H., Ishii K., Nagai S., Kakinuma H., Sasaki A., Yoshioka K., Kuramoto T., Shiono Y., Funao H., Isogai N. (2021). An Antibacterial Coated Polymer Prevents Biofilm Formation and Implant-Associated Infection. Sci. Rep..

[19] Hosseini Hooshiar M., Badkoobeh A., Kolahdouz S., Tadayonfard A., Mozaffari A., Nasiri K., Salari S., Safaralizadeh R., Yasamineh S. (2024). The Potential Use of Nanozymes as an Antibacterial Agents in Oral Infection, Periodontitis, and Peri-Implantitis. J. Nanobiotechnology.

[20] Lambiase F., Paoletti A., Grossi V., Di Ilio A. (2019). Analysis of Loads, Temperatures and Welds Morphology in FSW of Polycarbonate. J. Mater. Process. Technol..

[21] Eslami S., Tavares P.J., Moreira P.M.G.P. (2017). Friction Stir Welding Tooling for Polymers: Review and Prospects. Int. J. Adv. Manuf. Technol..

[22] Payganeh G.H., Mostafa Arab N.B., Dadgar Asl Y., Ghasemi F.A., Saeidi Boroujeni M. (2011). Effects of Friction Stir Welding Process Parameters on Appearance and Strength of Polypropylene Composite Welds. Int. J. Phys. Sci..

[23] Aghajani Derazkola H., Simchi A., Lambiase F. (2019). Friction Stir Welding of Polycarbonate Lap Joints: Relationship between Processing Parameters and Mechanical Properties. Polym. Test..

[24] Squeo E.A., Bruno G., Guglielmotti A., Quadrini F. (2009). Friction Stir Welding of Polyethylene Sheets. Annals of ”Dunarea de Jos” University of Galati.

[25] Vijendra B., Sharma A. (2015). Induction Heated Tool Assisted Friction-Stir Welding (i-FSW): A Novel Hybrid Process for Joining of Thermoplastics. J. Manuf. Process..

[26] Banjare P.N., Sahlot P., Arora A. (2017). An Assisted Heating Tool Design for FSW of Thermoplastics. J. Mater. Process. Technol..

[27] Aydin M. (2010). Effects of Welding Parameters and Pre-Heating on the Friction Stir Welding of UHMW-Polyethylene. Polym. Plast. Technol. Eng..

[28] Kiss Z., Czigány T. (2012). Microscopic Analysis of the Morphology of Seams in Friction Stir Welded Polypropylene. Express Polym. Lett..

[29] Pirizadeh M., Azdast T., Rash Ahmadi S., Mamaghani Shishavan S., Bagheri A. (2014). Friction Stir Welding of Thermoplastics Using a Newly Designed Tool. Mater. Des..

[30] Simoes F., Rodrigues D.M. (2014). Material Flow and Thermo-Mechanical Conditions during Friction Stir Welding of Polymers: Literature Review, Experimental Results and Empirical Analysis. Mater. Des..

[31] Kumar R., Singh R., Ahuja I.P.S., Penna R., Feo L. (2018). Weldability of Thermoplastic Materials for Friction Stir Welding- A State of Art Review and Future Applications. Compos. Part B Eng..

[32] Huang Y., Meng X., Xie Y., Wan L., Lv Z., Cao J., Feng J. (2018). Friction Stir Welding/Processing of Polymers and Polymer Matrix Composites. Compos. Part A Appl. Sci. Manuf..

[33] Azarsa E., Mostafapour A. (2014). Experimental Investigation on Flexural Behavior of Friction Stir Welded High Density Polyethylene Sheets. J. Manuf. Process..

[34] Mendes N., Loureiro A., Martins C., Neto P., Pires J.N. (2014). Morphology and Strength of Acrylonitrile Butadiene Styrene Welds Performed by Robotic Friction Stir Welding. Mater. Des..

[35] Bagheri A., Azdast T., Doniavi A. (2013). An Experimental Study on Mechanical Properties of Friction Stir Welded ABS Sheets. Mater. Des..

[36] Sharma A.K.R., Roy Choudhury M., Debnath K. (2020). Experimental Investigation of Friction Stir Welding of PLA. Weld. World.

[37] Zafar A., Awang M., Khan S.R. (2018). 3rd International Conference on Mechanical, Manufacturing and Process Plant Engineering (ICMMPE 2017). IOP Conf. Ser. Mater. Sci. Eng..

[38] Bajakke P.A., Malik V.R., Deshpande A.S. (2019). Particulate Metal Matrix Composites and Their Fabrication via Friction Stir Processing–a Review. Mater. Manuf. Process..

[39] Nath R.K., Maji P., Barma J.D. (2021). Joining of Advance Engineering Thermoplastic Using Novel Self-Heated FSW Tool. Jom.

[40] Jose J.V., Panneerselvam K. (2020). Joining of PEEK Plates by Friction Stir Welding Process. Mater. Today Proc..

[41] Gagliardi F., Ambrogio G., Conte R., Russo P. (2021). Investigation of Friction Stir Forming for Mechanical Interlocking High-Performance Polymers and Aluminium Sheets. Manuf. Lett..

[42] Khadka P., Varglund S., Akinwamide S., Vilaça P. (2023). Characterization of Friction Stir—Based Linear Continuous Joining of Aluminium Alloy to Structural Polymer. Weld. World.

[43] Huang Y., Meng X., Wang Y., Xie Y., Zhou L. (2018). Joining of Aluminum Alloy and Polymer via Friction Stir Lap Welding. J. Mater. Process. Technol..

[44] Dong H., Tang Z., Li P., Wu B., Hao X., Ma C. (2021). Friction Stir Spot Welding of 5052 Aluminum Alloy to Carbon Fiber Reinforced Polyether Ether Ketone Composites. Mater. Des..

[45] Huang Y., Meng X., Xie Y., Li J., Wan L. (2018). Joining of Carbon Fiber Reinforced Thermoplastic and Metal via Friction Stir Welding with Co-Controlling Shape and Performance. Compos. Part A Appl. Sci. Manuf..

[46] Li J., Dong H., Tang Z., Li P., Wu B., Ma Y., Huang L., Zhang L., Li C., Xiong J. (2023). Influence of Surface Pretreatment on the Bonding Mechanism and Mechanical Properties of AA5052/CFRP Friction Stir Spot Welded Joint. J. Manuf. Process..

[47] Li M., Xiong X., Ji S., Hu W., Yue Y. (2021). Achieving High-Quality Metal to Polymer-Matrix Composites Joint via Top-Thermic Solid-State Lap Joining. Compos. Part B Eng..

[48] Almasi D., Lau W.J., Rasaee S., Sharifi R., Mozaffari H.R. (2020). Fabrication of a Novel Hydroxyapatite/Polyether Ether Ketone Surface Nanocomposite via Friction Stir Processing for Orthopedic and Dental Applications. Prog. Biomater..

[49] Almasi D., Lau W.J., Rasaee S., Abbasi K. (2022). Fabrication and in Vitro Study of 3D Novel Porous Hydroxyapatite/Polyether Ether Ketone Surface Nanocomposite. J. Biomed. Mater. Res. Part B Appl. Biomater..

[50] Vidakis N., Petousis M., Mountakis N., Kechagias J.D. (2022). Material Extrusion 3D Printing and Friction Stir Welding: An Insight into the Weldability of Polylactic Acid Plates Based on a Full Factorial Design. Int. J. Adv. Manuf. Technol..

[51] Parast M.S.A., Bagheri A., Kami A., Azadi M., Asghari V. (2022). Bending Fatigue Behavior of Fused Filament Fabrication 3D-Printed ABS and PLA Joints with Rotary Friction Welding. Prog. Addit. Manuf..

[52] Mendes N., Loureiro A., Martins C., Neto P., Pires J.N. (2014). Effect of Friction Stir Welding Parameters on Morphology and Strength of Acrylonitrile Butadiene Styrene Plate Welds. Mater. Des..

[53] Arici A., Mert Ş. (2008). Friction Stir Spot Welding of Polypropylene. J. Reinf. Plast. Compos..

[54] Bilici M.K. (2012). Application of Taguchi Approach to Optimize Friction Stir Spot Welding Parameters of Polypropylene. Mater. Des..

[55] Bilici M.K., Yükler A.I., Kurtulmuş M. (2011). The Optimization of Welding Parameters for Friction Stir Spot Welding of High Density Polyethylene Sheets. Mater. Des..

[56] Verma S., Misra J.P. (2017). Study on Temperature Distribution during Friction Stir Welding of 6082 Aluminum Alloy. Mater. Today Proc..

[57] Senthil S.M., Kumar M.B. (2021). Effect of Tool Rotational Speed and Traverse Speed on Friction Stir Welding of 3D-Printed Polylactic Acid Material. Appl. Sci. Eng. Prog..

[58] Apium PEEK 450 Natural Datasheet. https://apiumtec.com/en/case-studies-datasheets.

[59] Gupta H., Salovey R. (1990). Thermal Behavior of Transparent Poly(Etheretherketone)(PEEK) Film. Polym. Eng. Sci..

[60] Regis M., Bellare A., Pascolini T., Bracco P. (2017). Characterization of Thermally Annealed PEEK and CFR-PEEK Composites: Structure-Properties Relationships. Polym. Degrad. Stab..

[61] Bonmatin M., Chabert F., Bernhart G., Djilali T. (2021). Rheological and Crystallization Behaviors of Low Processing Temperature Poly(Aryl Ether Ketone). J. Appl. Polym. Sci..

[62] Blundell D.J., Osborn B.N. (1983). The Morphology of Poly(Aryl-Ether-Ether-Ketone). Polymer.

[63] Chowdhury S.M., Chen D.L., Bhole S.D., Cao X. (2010). Tensile Properties of a Friction Stir Welded Magnesium Alloy: Effect of Pin Tool Thread Orientation and Weld Pitch. Mater. Sci. Eng. A.

[64] Panneerselvam K., Lenin K. (2014). Joining of Nylon 6 Plate by Friction Stir Welding Process Using Threaded Pin Profile. Mater. Des..

[65] Zhang H.W., Zhang Z., Chen J.T. (2005). The Finite Element Simulation of the Friction Stir Welding Process. Mater. Sci. Eng. A.

[66] Tej B., Sudeep C., Gummadi K., Elhattab K., Ahlstrom J., Sikder P. (2022). In—House Processing of 3D Printable Polyetheretherketone ( PEEK ) Filaments and the Effect of Fused Deposition Modeling Parameters on 3D—Printed PEEK Structures. Int. J. Adv. Manuf. Technol..

[67] Talbott M.F., Springer G.S., Berglund L.A. (1987). The Effects of Crystallinity on the Mechanical Properties of PEEK Polymer and Graphite Fiber Reinforced PEEK. J. Compos. Mater..

[68] Naffakh M., Gómez M.A., Ellis G., Marco C. (2003). Thermal Properties, Structure and Morphology of PEEK/Thermotropic Liquid Crystalline Polymer Blends. Polym. Int..

[69] Rinaldi M., Ghidini T., Cecchini F., Brandao A., Nanni F. (2018). Additive Layer Manufacturing of Poly (Ether Ether Ketone) via FDM. Compos. Part B Eng..

